# Glutaminolysis regulates endometrial fibrosis in intrauterine adhesion via modulating mitochondrial function

**DOI:** 10.1186/s40659-024-00492-3

**Published:** 2024-04-01

**Authors:** Pei Chen, Chaoshuang Ye, Yunke Huang, Bingning Xu, Tianyu Wu, Yuanhang Dong, Yang Jin, Li Zhao, Changchang Hu, Jingxia Mao, Ruijin Wu

**Affiliations:** 1 Department of Obstetrics and Gynecology, Women’s Hospital, Zhejiang University School of Medicine, Hangzhou, China; 2 Key Laboratory of Women’s Reproductive Health of Zhejiang Province, Hangzhou, China

**Keywords:** Glutaminolysis, Mitochondria, Endometrial stromal cell, Endometrial fibrosis, Intrauterine adhesion

## Abstract

**Background:**

Endometrial fibrosis, a significant characteristic of intrauterine adhesion (IUA), is caused by the excessive differentiation and activation of endometrial stromal cells (ESCs). Glutaminolysis is the metabolic process of glutamine (Gln), which has been implicated in multiple types of organ fibrosis. So far, little is known about whether glutaminolysis plays a role in endometrial fibrosis.

**Methods:**

The activation model of ESCs was constructed by TGF-β1, followed by RNA-sequencing analysis. Changes in glutaminase1 (GLS1) expression at RNA and protein levels in activated ESCs were verified experimentally. Human IUA samples were collected to verify GLS1 expression in endometrial fibrosis. GLS1 inhibitor and glutamine deprivation were applied to ESCs models to investigate the biological functions and mechanisms of glutaminolysis in ESCs activation. The IUA mice model was established to explore the effect of glutaminolysis inhibition on endometrial fibrosis.

**Results:**

We found that GLS1 expression was significantly increased in activated ESCs models and fibrotic endometrium. Glutaminolysis inhibition by GLS1 inhibitor bis-2-(5-phenylacetamido-1,2,4-thiadiazol-2-yl) ethyl sulfide (BPTES or glutamine deprivation treatment suppressed the expression of two fibrotic markers, α-SMA and collagen I, as well as the mitochondrial function and mTORC1 signaling in ESCs. Furthermore, inhibition of the mTORC1 signaling pathway by rapamycin suppressed ESCs activation. In IUA mice models, BPTES treatment significantly ameliorated endometrial fibrosis and improved pregnancy outcomes.

**Conclusion:**

Glutaminolysis and glutaminolysis-associated mTOR signaling play a role in the activation of ESCs and the pathogenesis of endometrial fibrosis through regulating mitochondrial function. Glutaminolysis inhibition suppresses the activation of ESCs, which might be a novel therapeutic strategy for IUA.

## Background

Intrauterine adhesion (IUA) is a disease characterized by partial or complete uterine cavity obliteration and is histologically characterized by endometrial fibrosis. IUA occurs when the endometrial basal layer is injured by intrauterine trauma, radiation, or infection, which usually induces hypomenorrhea, amenorrhea, and infertility [1, 2]. Because of the critical role of endometrial reception in embryo implantation, placenta, and development, IUA has become the second leading cause of female secondary infertility [3, 4]. Hysteroscopic adhesiolysis combined with postoperative hormone therapy and placement of intrauterine devices or other derivatization agents, including intrauterine suitable balloon and biogel, are currently the primary clinical interventions for IUA [5]. However, the common therapeutics have made no breakthrough to treat the severe fibrotic scarring due to the uncertain endometrial fibrosis mechanisms and the limitation to halt or reverse the progression of endometrial fibrosis. Encouragingly, recent studies found that transplantation of human amnion epithelial cells could inhibit endometrial fibrosis and improve endometrial regeneration in rodent IUA models [6, 7]. In-depth studies on underline fibrotic mechanisms and potential targets are imperative and might benefit the development of anti-fibrotic therapy for intrauterine adhesions.

The major pathological changes of fibrosis manifest as the replacement of normal stroma and glands with fibrous tissue, spindle-shaped myofibroblasts and inactive endometrial epithelium [8], as well as the excessive deposition of extracellular matrix (ECM) [9]. Fibrosis is considered an abnormal wound healing process, during which the persistent abnormal activation of myofibroblasts plays a key effector role. Myofibroblasts originate from resident stromal cells [4, 10, 11]. After activation, myofibroblasts exhibit a myofibroblastic phenotype expressing alpha-smooth muscle actin (α-SMA) and producing a large number of ECM proteins such as Collagen I [12, 13]. Activated monocytes and macrophages release inflammatory mediators that promote myofibroblasts accumulation and ultimately fibrosis [14, 15]. The high expression of TGF-β1, TNF-α, IFN-γ, IL-6, and IL-18 has been found in IUA endometrium [16–18]. Among these mediators, TGF-β1 is considered an essential pro-fibrotic factor in IUA and has been used to establish the activation model from endometrial stromal cells (ESCs) to myofibroblasts for studying endometrial fibrosis [19, 20].

Metabolic reprogramming, including dysregulation of glucose, lipid and glutamine metabolism, has been proposed to be a characteristic and contributor to fibrosis [21–23]. Glutamine metabolism (glutaminolysis) is required for TGF-β1-induced differentiation and activation of lung myofibroblast [24]. Hypoxia-inducible factor-1α (HIF-1α) metabolically controls collagen synthesis and modification in chondrocytes, depending on glutaminolysis-derived α-ketoglutarate (α-KG) [25]. Glutaminolysis starts in the mitochondria as a two-step enzymatic reaction that first converts glutamine to glutamate by glutaminase (GLS), and then converts glutamate (Glu) to α-KG by either transaminase or glutamate dehydrogenase [26]. Glutaminase is the key rate-limiting enzyme of glutaminolysis, including GLS1 and GLS2 [27]. Mitochondria are the main sites of glutamine metabolism, and their morphological and functional changes are closely related to glutamine metabolism. Low glutamine levels have been reported to cause mitochondrial elongation by diluting damaged mitochondrial proteins [28]. Furthermore, glutaminolysis is involved in mitochondrial biosynthesis by supplying carbon and nitrogen source of macromolecule and other metabolites, including amino acids and ammonia [29]. Glutaminolysis might also participate in mitochondrial energy metabolism by supplementing α-KG to enhance the TCA cycle. Besides, glutaminolysis could interact with the mammalian target of rapamycin (mTOR) complex 1 (mTORC1) to regulate protein biosynthesis and T-cell differentiation [29, 30]. It has been recently reported that mTORC1 mediates the expression of nuclear-encoded mitochondria-related genes at transcription and translation levels to regulate mitochondrial biogenesis and energy production [31, 32]. Aberrant activation of mTOR pathway has been observed in renal fibrosis of lupus nephritis and lung fibroblasts [33–35].

However, it remains unclear whether glutaminolysis and glutaminolysis-associated mTOR pathways play a role in the activation of ESCs and the pathogenesis of endometrial fibrosis. Research on glutaminolysis-related fibrosis mechanism, as well as the mitochondrial ultrastructure and respiratory function, would help us to better understand the complex pathogenesis of IUA. This study focuses on the role of glutaminolysis in mitochondrial function and how glutaminolysis functions in endometrial fibrosis, and attempts to explore the preclinical efficacy of glutaminolysis inhibition via mice IUA models.

## Results

### GLS1 expression was increased in TGF-β1-treated ESCs

**Fig. 1:**
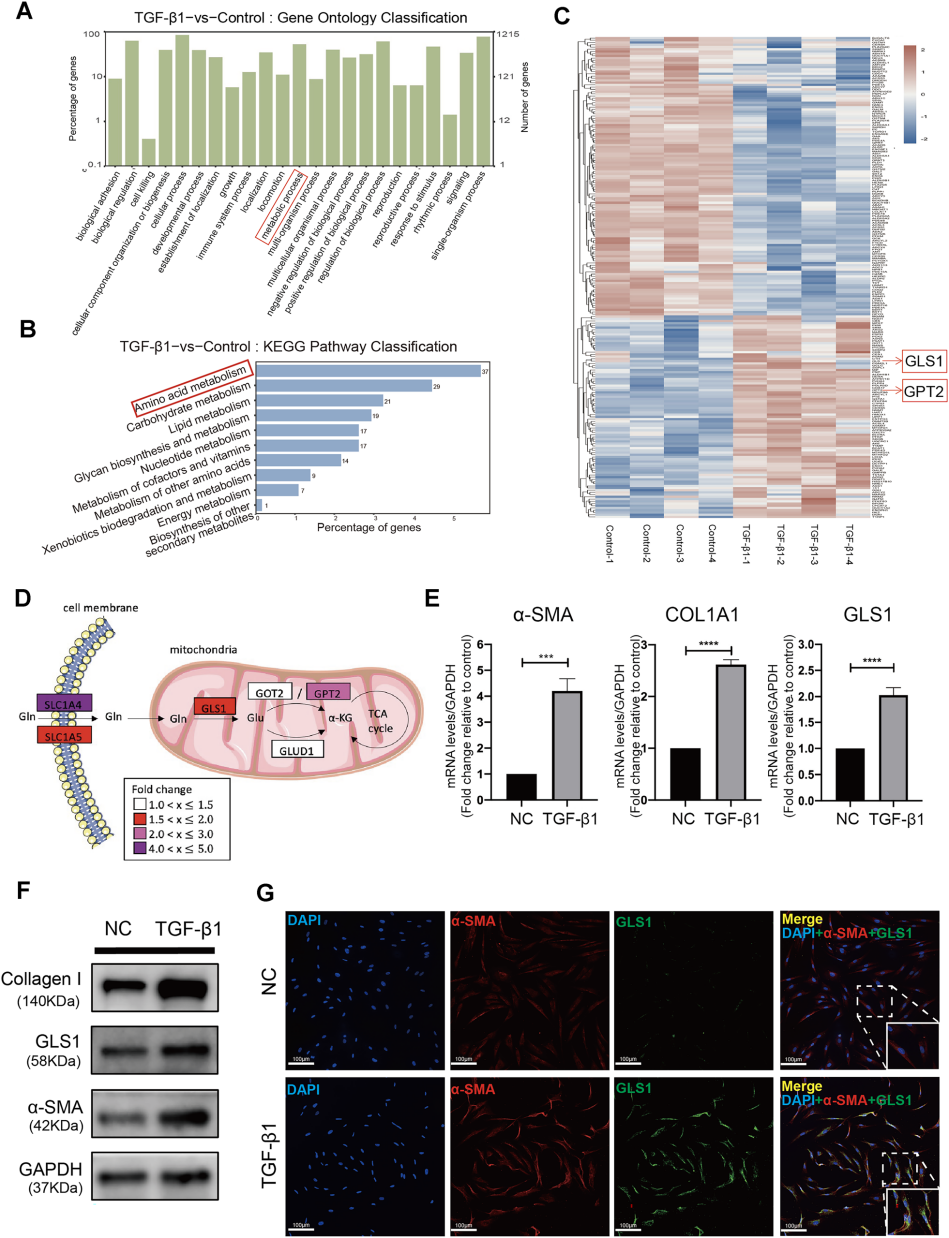
GLS1 expression was increased in TGF-β1-treated ESCs. ESCs were treated with or without 10 ng/ml TGF-β1 for 48 h. **A**–**D** Analysis of RNA sequencing data. *n* = 4 in each group. **A** Bar plot of GO analysis of the up-regulated differentially expressed genes in terms of biological process. **B** Bar plot of KEGG pathway classification of the up-regulated differentially expressed genes in terms of metabolism category. **C** Heat map of metabolism-related genes. **D** Schematic diagrams of glutaminolysis and the changes in mRNA abundance of genes in the indicated glutaminolysis pathways. **E** mRNA level of fibrotic markers (α-SMA, Collagen I) and GLS1 in ESCs after TGF-β1 treatment by quantitative PCR analysis for the validation of mRNA sequencing. *n* = 8 in each group. **F** protein levels of α-SMA, Collagen I and GLS1 in TGF-β1-treated ESCs by Western Blot analysis. **G** Representative immunocytochemistry of α-SMA, GLS1 and DAPI staining in ESCs after TGF-β1 treatment. Scale Bars = 100 μm. Statistical analyses were performed using Student’s t-test. ***p < 0.001. ****p < 0.0001

To investigate the possible mechanisms of ESCs activation, TGF-β1 was used to construct a model of ESCs activation [19, 20]. We used RNA sequencing to explore gene expression profiles of ESCs treated with or without TGF-β1. A total of 3556 genes were significantly differentially expressed in TGF-β1-treated ESCs, including 1576 up-regulated genes and 1980 down-regulated genes (adjusted p value < 0.05, |Fold Change| ≥ 1.5). GO terms at the second level were used for the GO annotation to illustrate general functional categories [36, 37], and the results showed that in terms of biological process, the up-regulated DEGs were enriched in metabolic process (Fig. 1A). Further KEGG pathway classification revealed that the up-regulated DEGs were mainly involved in amino acid metabolism in terms of metabolism category (Fig. 1B).

Glutamine is the most abundant amino acid found in mammals. TGF-β1 treatment up-regulated the gene expression level of glutaminolysis-related enzymes GLS1 and GPT2, as shown by heatmap analysis of metabolism-related genes (Fig. 1C). Glutamine is taken into cells by transporters SLC1A4 or SLC1A5. Then glutamine is converted to glutamate in mitochondria by GLS1, the key rate-limiting enzyme of glutaminolysis, and further metabolized to α-KG via glutamate dehydrogenase GLUD1 or transaminases such as glutamate-oxaloacetate transaminase 2 (GOT2) or glutamate-pyruvate transaminase 2 (GPT2) (Fig. 1D). Compared with TGF-β1-untreated ESCs, mRNA expression of α-SMA, Collagen I and GLS1 were increased in TGF-β1-treated ESCs (*p* < 0.001, *p* < 0.0001, *p* < 0.0001, respectively; Fig. 1E). The increased expression of α-SMA, Collagen I and GLS1 were further validated at protein levels (Fig. 1F). Co-immunofluorescence microscopy showed co-localization of GLS1 with α-SMA in TGF-β1-treated activated ESCs (Fig. 1G).

### GLS1 expression was increased in the fibrotic endometrium of IUA patients

**Fig. 2:**
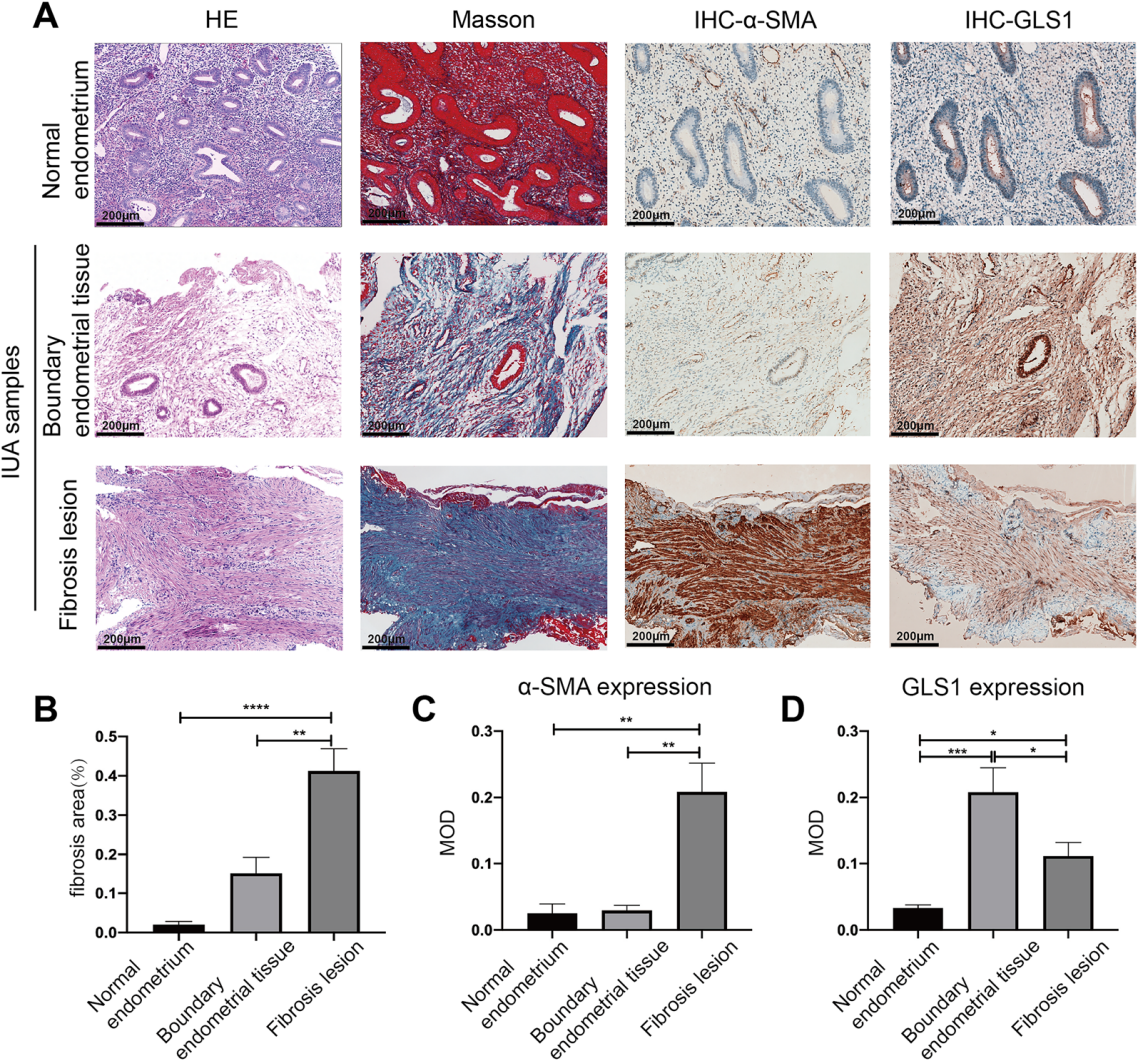
GLS1 expression was increased in the fibrotic endometrium of IUA patients. **A** Representative HE staining, Masson staining, α-SMA IHC staining and GLS1 IHC staining of sections from normal endometrium and IUA samples with different stages of fibrosis (including dense fibrous lesions and borderline fibrosis lesions). Scale Bars = 200 μm. **B**–**D** Quantitative analysis of the percentage of the blue areas of Masson staining (**B**), and the mean optical density (MOD) of α-SMA staining (**C**) and GLS1 staining (**D**) in each group. All data were obtained from five normal endometrial patients and five IUA patients. Statistical analyses were performed by one-way ANOVA plus Tukey’s multiple comparisons test. *p < 0.05, **p < 0.01, ***p < 0.001, ****p < 0.0001

To verify our findings of GLS1 expression in ESCs, we next extended our experiments to clinical IUA endometrial samples. HE staining revealed the replacement of endometrial stroma by dense fibrous tissue with a decrease or a disappearance of glands in fibrosis lesions of IUA patients (Fig. 2A). Masson trichrome staining showed significantly more collagen deposition in fibrous lesions than that in normal endometrium, as indicated by the blue area percentages of the endometrium (*p* < 0.0001; Fig. 2A, B). IHC staining showed that the expression of α-SMA and GLS1 was higher in the fibrous lesions than that in normal endometrial stroma (*p* < 0.01, *p* < 0.05, respectively; Fig. 2A, C, D). It was interesting to find borderline fibrotic regions in which dense fibrous tissues had not yet completely formed. Comparing with normal endometrium, there was a trend of increased collagen deposition in borderline regions, although the difference was not statistically significant, and there are no differences in α-SMA expression (Fig. 2A–C). Moreover, the expression of GLS1 was significantly higher in the borderline regions than that in normal endometrial stroma (*p* < 0.001; Fig. 2A, D).

### Inhibition of glutaminolysis suppressed α-SMA and Collagen I expression in TGF-β1–induced ESCs

**Fig. 3:**
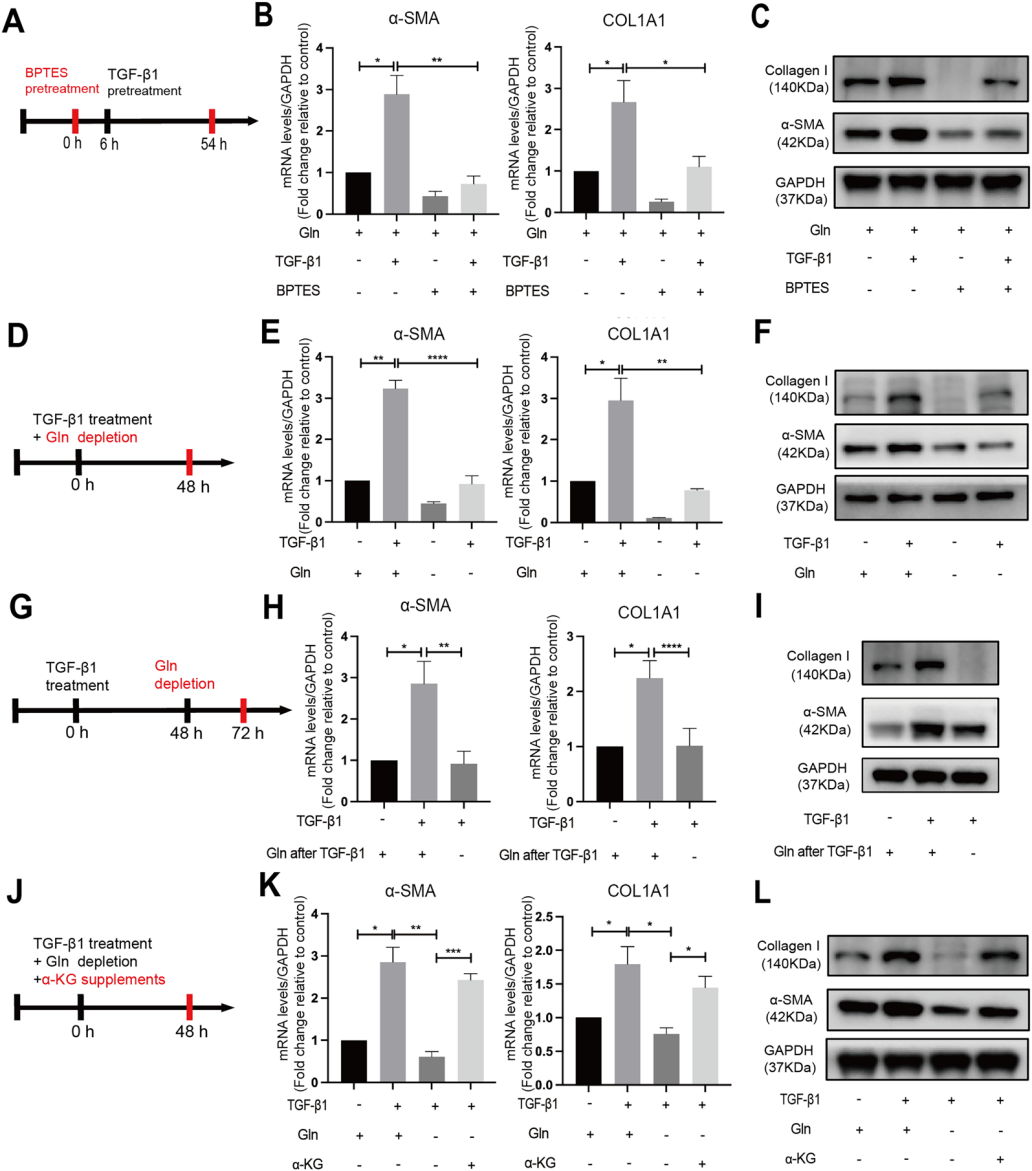
Inhibition of glutaminolysis suppressed α-SMA and Collagen I expression in TGF-β1–induced ESCs. **A**–**C** ESCs were treated with vehicle (0.1% DMSO) or BPTES (10 µM) for 6 h and then treated together with or without 10 ng/ml TGF-β1 for 48 h (**A**). The changes in α-SMA and Collagen I mRNA levels were determined by quantitative PCR analysis (**B**), changes in α-SMA and Collagen I protein levels were determined by Western Blot analysis (**C**). **D**–**F** ESCs were treated with or without 10 ng/ml TGF-β1 in the presence or absence of 2 mM glutamine for 48 h (**D**). The changes in mRNA levels (**E**) and protein levels (**F**) of α-SMA and Collagen I were determined. **G-I** ESCs were treated with or without 10 ng/ml TGF-β1 for 48 h and switched to Gln-free or Gln-containing medium for 24 h after TGF-β1 treatment (**G**). The changes in mRNA levels (**H**) and protein levels (**I**) of α-SMA and Collagen I were determined. **J**–**L** ESCs were treated with 10 ng/ml TGF-β1 in the presence or absence of 2 mM Gln or 2 mM α-KG for 48 h (**J**). The changes in mRNA levels (**K**) and protein levels (**L**) were determined. Treatment groups compared with their control (set at 1) was analyzed using a one-sample Student’s t-test, and subsequent comparisons of treatment groups was performed using the parametric analysis (student’s t test or ANOVA followed by Turkey post-hoc analysis). *p < 0.05, **p < 0.01, ***p < 0.001, ****p < 0.0001. *n* = 4 in each group

To investigate whether the changes of glutaminolysis act on ESCs activation, we used the potent GLS1 inhibitor BPTES in ESCs, and conducted glutamine deprivation tests and rescue assays. 10 µM BPTES pretreatment before TGF-β1 stimulation (Fig. 3A) caused a reduction in TGF-β1–induced expression of α-SMA and Collagen I at the mRNA levels (Fig. 3B) and protein levels (Fig. 3C), indicating that GLS1-mediated glutaminolysis was involved in the activation of ESCs. Consistently, the deprivation of glutamine from the culture medium during TGF-β1 treatment (Fig. 3D) suppressed the expression of α-SMA and Collagen I (Fig. 3E, F). We next explored the effect of glutamine deprivation after TGF-β1 treatment on the expression of α-SMA and Collagen I. ESCs were switched from Gln-containing medium to Gln-free medium at the end of TGF-β1 stimulation (Fig. 3G), leading to a decline in the mRNA and protein expression levels of α-SMA and Collagen I (Fig. 3H, I). Supplementing the glutamine-free medium with 2 mM α-KG (Fig. 3J), a downstream metabolite of glutaminolysis, rescued the decreased α-SMA and Collagen I mRNA and protein expression induced by glutamine depletion (Fig. 3K, L).

### Inhibition of glutaminolysis alleviated endometrial fibrosis in the IUA mice model

**Fig. 4:**
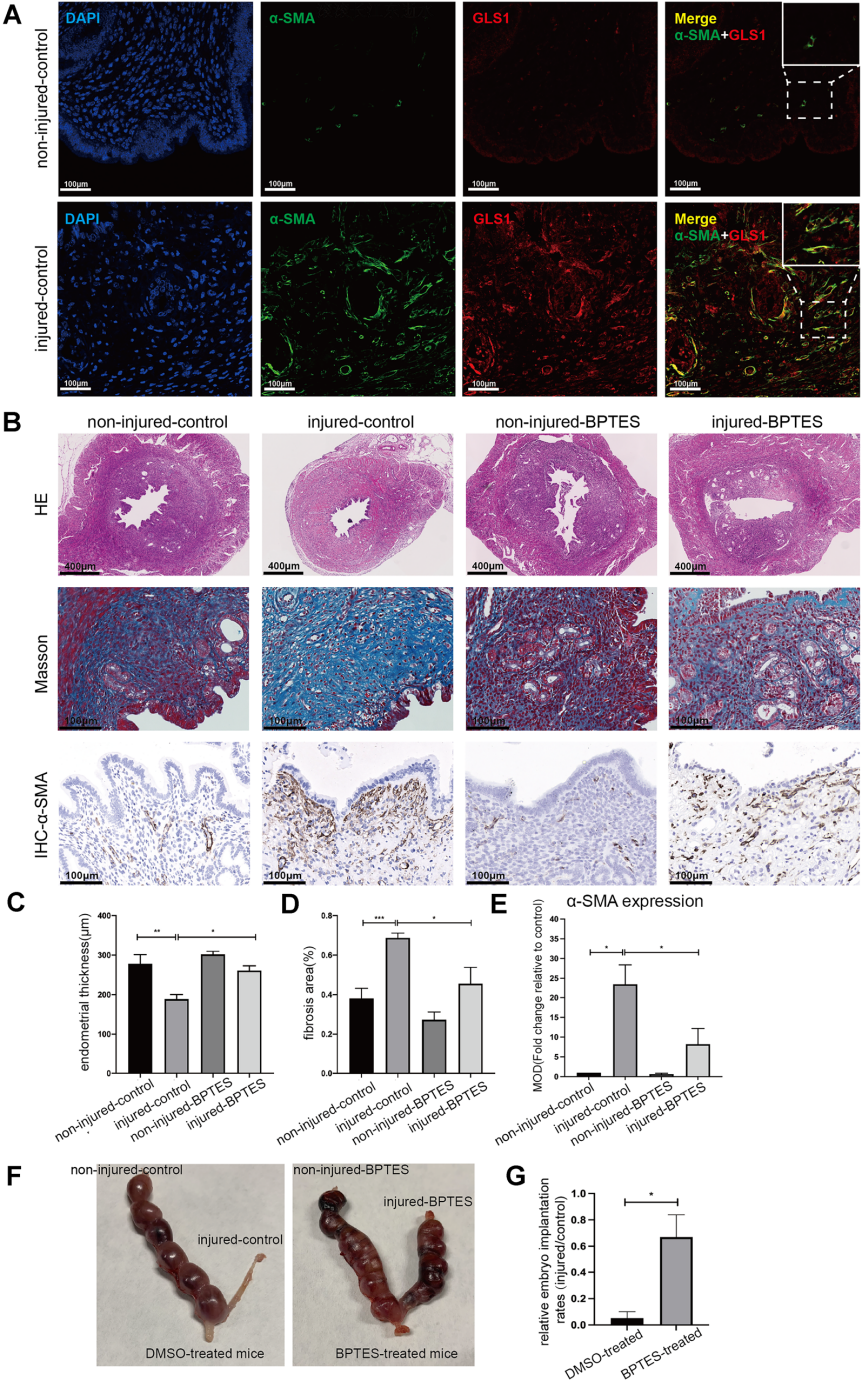
Inhibition of glutaminolysis alleviated endometrial fibrosis in the IUA mice model. The uteri of mice were divided into 4 groups: non-injured-control represented uninjured uterus from DMSO-treated mice; injured-control represented injured uterus from DMSO-treated mice; non-injured-BPTES represented uninjured uterus from BPTES-treated mice; injured-BPTES represented injured uterus from BPTES-treated mice. **A** Representative α-SMA and GLS1 co-immunofluorescence-stained transverse uterine sections of IUA model mice. Scale Bars = 100 μm. **B** Representative images of HE staining (Scale Bars = 400 μm), Masson staining (Scale Bars = 100 μm), and α-SMA IHC staining (Scale Bars = 100 μm) of uterine transverse sections from each group. **C**–**E** Quantitative analysis of the average endometrial thickness (**C**), the percentage of the blue areas of Masson staining (**D**), and the MOD of α-SMA staining (**E**) in each group. *n* = 5 in each group. Statistical analyses were performed by one-way ANOVA plus Tukey’s multiple comparisons test. **F** Representative images of embryo implantation numbers in each group. **G** Quantitative analysis of the relative embryo implantation rates. *n* = 5 in each group. Statistical analyses were performed by Mann Whitney test. *p < 0.05, **p < 0.01, ***p < 0.001

To validate the role of glutaminolysis in the pathogenesis of endometrial fibrosis, we constructed a unilateral murine IUA model and further investigated the effects of the specific GLS1 inhibitor BPTES on fibrogenic response in vivo. Immunofluorescent staining showed the high co-localization of α-SMA with GLS1 in fibrotic endometrium (Fig. 4A), indicating that myofibroblastic ESCs upregulated GLS1.

Injured endometrium demonstrated characteristic histological features of endometrial fibrosis with the decreased endometrial thickness (*p* < 0.01; Fig. 4B, C). The degree of collagen deposition (*p* < 0.001; Fig. 4B, D), shown as blue area percentage, and the α-SMA expression (*p* < 0.05; Fig. 4B, E) were increased in ethanol-injured endometrium compared to uninjured endometrium. BPTES treatment reduced the changes in endometrial thickness (*p* < 0.05; Fig. 4B, C) of injured endometrium induced by ethanol treatment. Masson trichrome staining (*p* < 0.05; Fig. 4B, D) and α-SMA IHC staining (*p* < 0.05; Fig. 4B, E) suggested that BPTES reduced collagen deposition and myofibroblast activation in injured endometrium compared with DMSO treatment. In addition, BPTES also improved pregnancy outcomes, as shown by an increase in the relative embryo implantation rates (*p* < 0.05; Fig. 4F, G). These data revealed that inhibition of glutaminolysis reduced the accumulation of activated myofibroblastic ESCs and mitigated endometrial fibrosis in vivo.

### Alterations in mitochondrial ultrastructure and mitochondrial dynamics of TGF-β1-treated ESCs

**Fig. 5:**
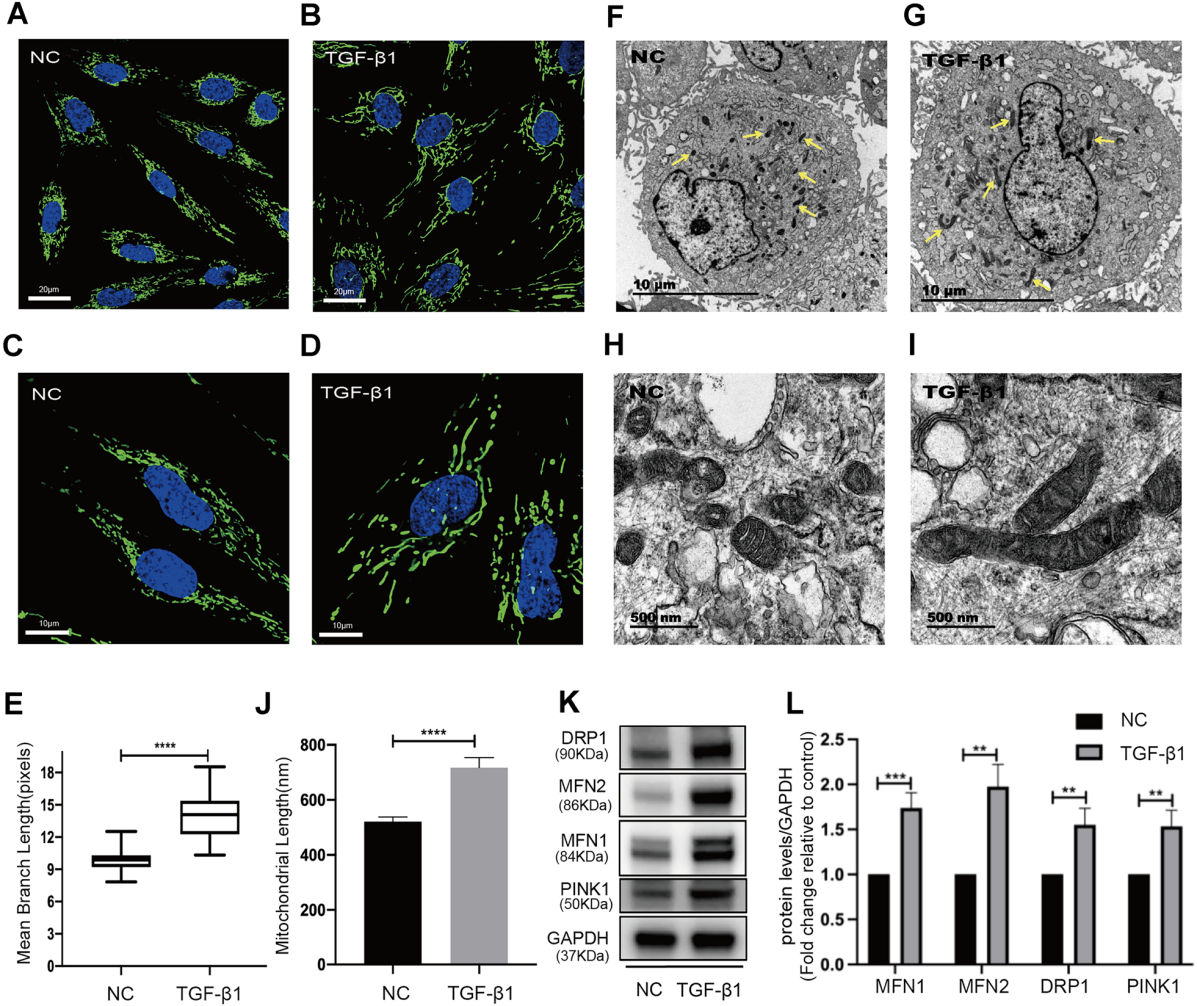
Mitochondria ultrastructure and the expression of proteins related to mitochondrial dynamics in TGF-β1-treated ESCs. **A**, **B** MitoTracker staining in ESCs treated without (**A**) or with (**B**) TGF-β1 (Scale Bars = 20 μm). **C**, **D** MitoTracker staining in ESCs treated without (**C**) or with (**D**) TGF-β1 (Scale Bars = 10 μm). **E** Quantitative analysis of mitochondrial mean branch length using the MINA toolset. 34 TGF-β1-untreated cells (NC) and 31 TGF-β1-treated cells from *n* = 3 independent experiments were blindly scored. Statistical analyses were performed by Student’s t-test. ****p < 0.0001. **F**, **G** TEM images of ESCs without (**F**) or with (**G**) TGF-β1 (Scale Bars = 10 μm). The yellow arrows indicate mitochondria. **H**, **I** TEM images of ESCs without (**H**) or with (**I**) TGF-β1 (Scale Bars = 500 nm). **J** Quantitative analysis of average mitochondrial major axis length. *n* = 3; 12–15 mitochondria/cell; 10 cells/per group. Statistical analyses were performed by Mann Whitney test. ****p < 0.0001. **K**, **L** Western Blot (**K**) and quantification analysis (**L**) of MFN1, MFN2, DRP1, and PINK1 protein levels in ESCs treated without or with TGF-β1 (*n* = 5 in each group). Statistical analyses were performed by using Student’s t-test. **p < 0.01, ***p < 0.001

ESCs treated with or without TGF-β1 were imaged using confocal and transmission electron microscopy (TEM) to assess mitochondrial morphology. It showed clear differences in mitochondrial morphology, as TGF-β1-treated ESCs exhibited a more fused and elongated mitochondrial phenotype than control cells (Fig. 5A–D). Quantification analysis of mitochondrial network morphology using the ImageJ macro tool MiNA showed that TGF-β1 treatment increased the mean branch length (*p* < 0.0001; Fig. 5E). Consistently, ultrastructural analysis by TEM showed mitochondria more elongated and tubular in TGF-β1-treated ESCs (Fig. 5F–I), confirmed by the quantification analysis of mitochondrial length from the TEM images (*p* < 0.0001; Fig. 5J). The maintenance of mitochondrial morphology depends on the dynamic balance among fusion, division and mitophagy. To better understand this phenomenon of mitochondrial elongation, we conducted Western Blot analysis of predominant proteins involved in mitochondrial dynamics, including mitofusin 1 (MFN1) and mitofusin 2 (MFN2) for fusion, dynamin-related protein 1 (DRP1) for fission, and PTEN-induced putative kinase 1 (PINK1) for mitophagy [38]. As shown in the Fig. 5K, L, the expression levels of MFN1, MFN2, DRP1, and PINK1 were all increased (*p* < 0.001, *p* < 0.01, *p* < 0.01, *p* < 0.01, respectively), suggesting an increase in fission, fusion, and mitophage events after TGF-β1 treatment. These findings indicated an imbalance in mitochondrial dynamics in the activated ESCs, ultimately manifesting as mitochondrial elongation.

### Effects of glutaminolysis on mitochondrial function

**Fig. 6:**
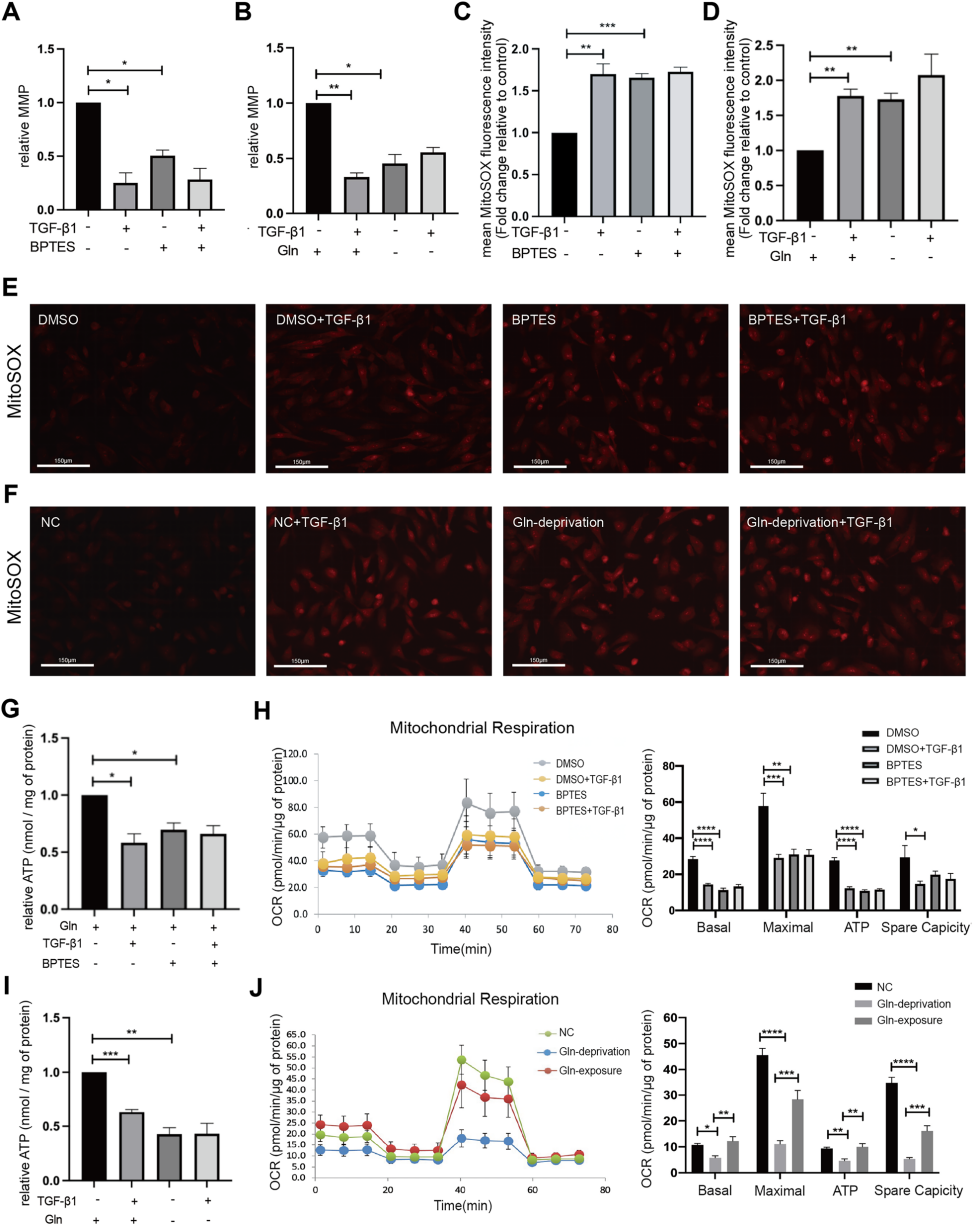
Analysis of mitochondrial function in glutaminolysis inhibition experiments. **A** Relative MMP analysis of ESCs treated with vehicle (0.1% DMSO) or BPTES (10 µM) for 6 h and then treated together with or without 10 ng/ml TGF-β1 for 48 h. **B** Relative MMP analysis of ESCs treated with or without 10 ng/ml TGF-β1 in the presence or absence of 2 mM glutamine for 48 h. **C** Relative MitoSOX fluorescence intensity of ESCs treated with or without 10 µM BPTES and 10 ng/ml TGF-β1. **D** Relative MitoSOX fluorescence intensity of ESCs treated with or without 2 mM glutamine and 10 ng/ml TGF-β1. **E**, **F** Representative images of MitoSOX staining in ESCs from each group. The scale bar = 150 μm. Data represent at least 3 biologically independent experiments. Treatment groups compared with their control (set at 1) was analyzed using a one-sample Student’s t-test, and subsequent comparisons of treatment groups was performed using the ANOVA followed by Turkey post-hoc analysis. **G** Relative mitochondrial ATP levels of ESCs treated with or without 10 µM BPTES and 10 ng/ml TGF-β1. **H** The OCR analysis of ESCs treated with or without 10 µM BPTES and 10 ng/ml TGF-β1. **I** Relative mitochondrial ATP levels of ESCs treated with or without 2 mM glutamine and 10 ng/ml TGF-β1. **J** The OCR analysis of ESCs cultured in Gln-containing or Gln-free medium for 48 h. To measure the acute response of ESCs to glutamine, ESCs were cultured in Gln-free medium, and glutamine was added back when OCR was measured. Data represent at least 3 biologically independent experiments. Statistical analyses were performed by one-way ANOVA plus Tukey’s multiple comparisons test. *p < 0.05, **p < 0.01, ***p < 0.001, ****p < 0.0001

To further investigate whether glutaminolysis exerted effects on mitochondrial function, we measured mitochondrial membrane potential (MMP, ΔΨm), mitochondrial reactive oxygen species (ROS) levels, mitochondrial ATP production and oxygen consumption rate (OCR) to evaluate the changes of mitochondrial function. ESCs treated with 10 µM GLS1 inhibitors BPTES and without TGF-β1 showed lower MMP compared to ESCs treated without BPTES and TGF-β1 (Fig. 6A). Glutamine deprivation caused a reduction in MMP of ESCs without TGF-β1 treatment (Fig. 6B). According to the MitoSOX assay, TGF-β1 treatment induced a remarkable elevation of mitochondrial ROS levels, and both BPTES treatment (Fig. 6C, E) and glutamine deprivation (Fig. 6D, F) could increase mitochondrial ROS levels, indicating an occurrence of excessive oxidative stress. Additionally, TGF-β1 treatment reduced mitochondrial ATP production in ESCs. As shown in Fig. 6G, BPTES treatment resulted in a notable decline in mitochondrial ATP production. Consistent with the mitochondrial ATP measurements, BPTES treatment caused a significant decrease in the basal OCR, maximal OCR, and ATP-linked OCR of ESCs without TGF-β1 treatment (Fig. 6H). Furthermore, glutamine deprivation also contributed to a decrease in mitochondrial ATP production in ESCs (Fig. 6I). ESCs cultured in medium lacking glutamine showed a significant reduction of the level of basal OCR, maximal OCR, ATP-linked OCR and spare capacity (Fig. 6J). After glutamine deprivation for 48 h, acute exposure of ESCs to glutamine dramatically increased the OCR, indicating that glutamine was required for mitochondrial respiration function of ESCs (Fig. 6J).

### Glutaminolysis regulated ESCs activation via activated mTORC1 pathway

**Fig. 7:**
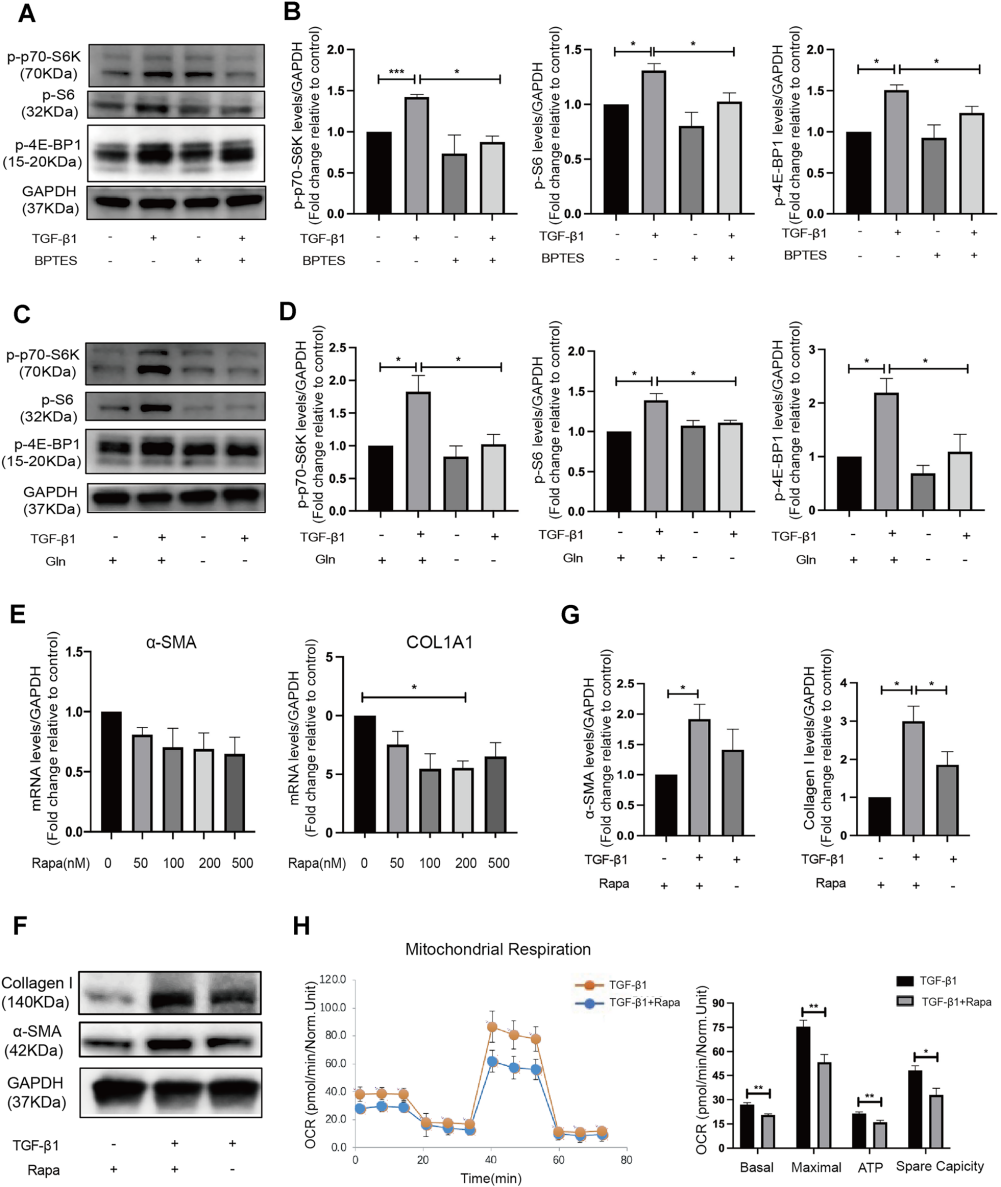
Glutaminolysis regulated ESCs activation via activated mTORC1 pathway. **A**, **B** Western blot (**A**) and quantification analysis (**B**) of p-p70-S6K, p-S6, p-4E-BP1 protein level in ESCs after pretreatment with BPTES for 6 h and TGF-β1 stimulation for another 48 h. **C**, **D** Western blot (**C**) and quantification analysis (**D**) of p-p70-S6K, p-S6, p-4E-BP1 protein level in ESCs treated with TGFβ1 in the presence or absence of glutamine for 48 h. **E** Quantitative PCR analysis of αSMA and Collagen I mRNA levels in ESCs after pretreatment with gradient doses of Rapamycin (Rapa, mTOR inhibitor) or 0.1% DMSO for 12 h and TGF-β1 stimulation for another 48 h. **F**, **G** Western blot (**F**) and quantification analysis (**G**) of α-SMA and Collagen I protein levels in ESCs pretreated with 200 nM Rapamycin or 0.1% DMSO for 12 h and treated with TGF-β1 stimulation for another 48 h. Treatment groups compared with their control (set at 1) was analyzed using a one-sample Student’s t-test, and subsequent comparisons of treatment groups was performed using the parametric analysis (Student’s t test or ANOVA followed by Turkey post-hoc analysis). **F** The OCR analysis of ESCs pretreated with vehicle (0.1% DMSO) or Rapa (200 nM) for 12 h and then treated together with 10 ng/ml TGF-β1 for 48 h (*n* = 5 in each group). Statistical analyses were performed by Student’s t-test. *p < 0.05, **p < 0.01, ***p < 0.001

The activation of mTORC1 was assessed by evaluating the phosphorylation of mTORC1 downstream targets, including S6, p70-S6K and 4E-BP1 [39]. We found increased phosphorylation levels of p70-S6K, S6, and 4E-BP1 in TGF-β1-treated ESCs, indicating up-regulated mTORC1 activity in activated myofibroblastic ESCs (Fig. 7A–D). 10 µM BPTES pretreatment for 6 h down-regulated the p-p70-S6K, p-S6 and p-4E-BP1 level in the presence of TGF-β1 (Fig. 7A, B). Consistent with this, removing glutamine from the culture medium during TGF-β1 stimulation also caused a decline in the phosphorylation of mTORC1 downstream targets (Fig. 7C, D). These results suggested that glutaminolysis enhanced mTORC1 signaling during the differentiation and activation of ESCs.

**Fig. 8:**
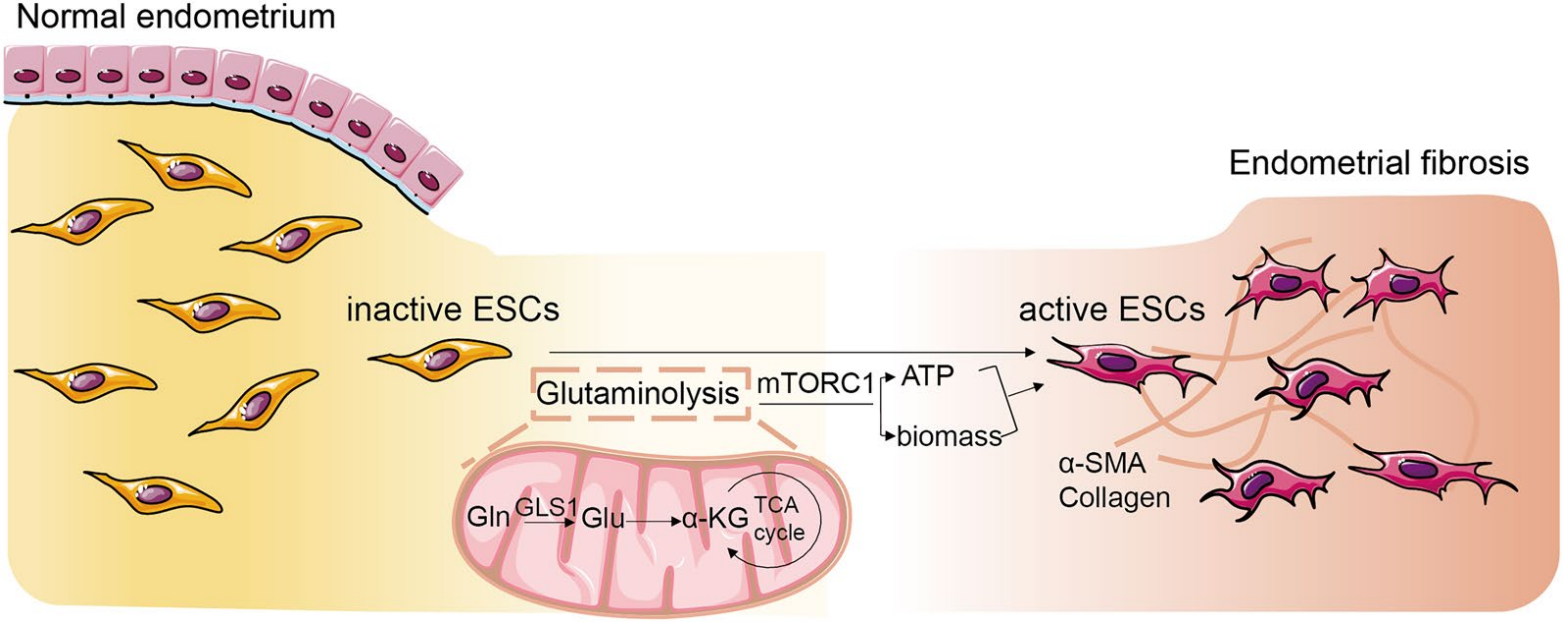
Schematic diagram of glutaminolysis in endometrial fibrosis. After damage, GLS1-mediated glutaminolysis is activated in endometrial stromal cells, causing a stroma-myofibroblast transition via mTOR pathway. Glutaminolysis participated in energy supply and maintenance of mitochondrial function during ESCs activation

Rapamycin (Rapa) is a potent mTOR inhibitor known to function by directly binding mTORC1. We suppressed the mTORC1 pathway to further investigate whether mTORC1 signaling pathway mediates the effect of glutaminolysis on ESCs activation. In TGF-β1-stimulated ESCs, 200 nM Rapa pretreatment for 12 h caused a decrease in Collagen I mRNA in ESCs treated with TGF-β1 (*p* < 0.5; Fig. 7E), which was paralleled by a corresponding reduction in protein expression (Fig. 7F, G). The expression of α-SMA did not show significant differences, but a declining tendency was observed (Fig. 7E–G). We then measured the effects of the mTORC1 pathway on mitochondrial respiration during ESCs activation. As shown in Fig. 7H, Rapamycin caused a significant decline in the basal OCR, maximal OCR, ATP-linked OCR and spare capacity of ESCs treated with TGF-β1.

## Discussion

IUA severely affects women’s menstruation and fertility. Treatments to prevent or reverse endometrial fibrosis in women of childbearing age susceptible to IUA or with IUA are great challenges for gynecologists and fertility specialists. Until now, efforts to develop such therapeutic solutions have proven unsuccessful because of the unclear mechanism. Theoretically, this could be accomplished by inhibiting the overactivation of myofibroblastic ESCs, the key effector cells of endometrial fibrosis. We focused on glutaminolysis and revealed how it contributes to regulating the myofibroblastic phenotypes and mitochondrial respiration functions of ESCs, exploring its potential mechanism as a therapeutic target for endometrial fibrosis in the future.

Alterations in glutaminolysis have been reported to play an essential role in cell fate and differentiation of multiple cell types. For instance, erythroid differentiation and Myc-induced transformation of cancer cells tend to be dependent on glutaminolysis [40, 41], glutaminolysis inhibition reduces Th17 differentiation of T cells from patients with Systemic Lupus Erythematosus (SLE) [42]. Our study is innovative for it provided evidence that glutaminolysis was involved in ESCs activation and differentiation. We found an increased expression of GLS1, the hallmark of glutaminolysis activation [23, 24, 43], in the activated ESCs model and fibrotic endometrium of IUA patients, accompanied by the increased α-SMA level and collagen deposition. In the process of pathologic fibrosis, the persistent presence and activation of myofibroblasts induce the expression of α-SMA, which is incorporated into stress fibers and promotes the contraction of ECM, maintaining the differentiated state in return [44, 45]. Collagen I, the most prevalent ECM component mainly produced by myofibroblasts, could increase ECM stiffness and exacerbate fibrosis [22]. In borderline fibrotic regions of endometrium, the protein expression levels of α-SMA did not show the same increasing trend as collagen deposition. This result indicates that collagen might be an earlier and more sensitive indicator of fibrosis than α-SMA, since the myofibroblast activation is a gradual maturation process [46, 47]. The expression of GLS1 was increased in the borderline fibrotic region, suggesting that the induction of glutaminolysis was involved in both the initial activation stage of ESCs and the further process of complete differentiation into myofibroblasts.

The therapeutic strategies aimed at interfering with myofibroblast activation, including small molecule inhibitors and monoclonal antibodies, have been proposed to inhibit fibrosis in the liver, lung, and kidneys [48–50]. In our study, inhibition of GLS1 activity by BPTES or deprivation of GLS1 substrate glutamine decreased the expression of the critical fibrotic markers α-SMA and Collagen I in ESCs. Consistent with our results, glutaminolysis regulated α-SMA and Collagen I expression in dermal fibroblasts and lung myofibroblasts [24, 51]. It was reported that glutamate and α-KG, the downstream metabolites of glutaminolysis, could provide a source of carbon and nitrogen directly or indirectly to promote the biosynthesis of glycine and proline, the two most important amino acids in collagen [52, 53]. Besides, α-KG-dependent histone demethylases upregulated the transcription of specific myofibroblast genes including α-SMA and COL1A1 [54]. These previous studies support our findings that supplementing exogenous α-KG could rescue the decreased expression of fibrotic markers induced by glutamine depletion, suggesting that glutaminolysis and its key metabolites participated in ESCs activation. Furthermore, the removal of glutamine post-activation effectively reversed the myofibroblast phenotype of ESCs. We also found that, in the case of glutamine deprivation post-TGF-β1-treatment, the reduction in collagen I protein levels were much more pronounced than the reduction in its mRNA levels. Post-translational modifications may contribute to this observation. It has been reported that α-KG could enhance collagen’s resistance to degradation by increasing proline and lysine hydroxylation on collagen [25]. Additionally, cells could convert collagen proteins into glutamine especially in conditions of nutrient deprivation [55]. Therefore, glutamine deprivation may reduce the stability of previously generated collagen proteins, resulting in more degradation and conversion. These results indicated the important role of glutaminolysis in maintaining the activated state of ESCs. Together, our findings provide a molecular target and theoretical basis for the preclinical animal researches. Thus, we further used BPTES to inhibit glutaminolysis in IUA mouse models. BPTES has been widely used in animal studies for multiple cancers and autoimmune diseases [42, 56]. In our study, BPTES treatment improved the morphology, receptivity and function of fibrotic endometrium to a certain extent, validating the utility of glutaminolysis inhibition for the treatment of endometrial fibrosis in vivo systems.

Acquisition and maintenance of a myofibroblast phenotype with a high synthetic ability for ECM proteins require energy. To mechanistically verify the contribution of glutaminolysis to endometrial fibrosis, we further investigated the relationship between glutaminolysis and mitochondria. Mitochondria are the essential cellular organelles linked to energy production whose morphology is closely associated with functional status. The complex interplay between mitochondrial function and TGF-β1 signaling remains inconclusive. Previous studies have shown that TGF-β1 was increased, accompanied by mitochondrial dysfunction in fibrosis tissues of dilated cardiomyopathy [57]. TGF-β1 exerted harmful effects on mitochondrial function by decreasing mitochondrial mass and mitochondrial DNA in human renal proximal tubular epithelial cells associated with renal fibrosis [58]. We also found decreased MMP, ATP production, and OCR of mitochondria, along with increased mitochondrial ROS levels in TGF-β1-activated ESCs, indicating excessive oxidative stress and mitochondrial dysfunction [59]. Additionally, we observed increased protein expressions of mitochondrial fusion, fission, and mitophagy in activated ESCs, concurrent with mitochondrial elongation, suggesting that mitochondrial elongation was the outcome of an imbalance in mitochondrial dynamics. In the case of mitochondrial dysfunction, damaged mitochondria would be cleared through mitochondrial fission and mitophagy [60]. However, under certain types of stress, including nutritional nutrient starvation and oxidative stress, cells may adaptively elongate mitochondria through mitochondrial fusion to dilute damaged mitochondrial proteins and enhance energetic efficiency [61, 62]. Several other studies have also observed mitochondrial elongation, along with active mitochondrial fission and fusion, which were associated with the maintenance of mitochondrial health and resistance to dysfunction [63, 64]. Therefore, we speculated that mitochondrial elongation corresponded to an event of fusion-dominated mitochondrial dynamic changes in ESCs, adapting to the energy shortages caused by TGF-β1 mediated mitochondrial dysfunction.

Studies have reported the increased dependence on glutaminolysis in cancer cells [65, 66] and neurons [67] with mitochondrial defects and high metabolic demands. In response to energy crisis or high anabolic demand, cells could cause an adaptive upregulation of glutaminolysis as a compensatory response [68, 69] to support the TCA cycle, providing reducing equivalent for oxidative phosphorylation or providing carbon source for biosynthesis [24, 70]. In our study, both GLS1 inhibition or glutamine deprivation could inhibit the mitochondrial functions of ESCs, while acute exposure to glutamine significantly promoted the OCR of ESCs, suggesting that glutamine was a critical energy substrate for maintaining mitochondrial respiratory function in ESCs during nutritional crisis. Consistent with our results, Smith et al. found that TGF-β1-induced activation of renal interstitial fibroblasts underwent metabolic reprogramming from oxidative phosphorylation to aerobic glycolysis and glutaminolysis to support energy production and biosynthesis [53, 71]. Combining published literature and our present results, it is reasonable to speculate that enhanced glutaminolysis acts as a compensatory response in ESCs with impaired mitochondrial function, supporting the activation of ESCs by maintaining mitochondrial energy supply.

mTOR signaling plays an essential role in energy homeostasis and metabolism. In our study, GLS1 expression was elevated together with mTORC1 signaling during ESCs activation, and glutaminolysis inhibition suppressed mTORC1 signaling, suggesting that the overexpression of GLS1 might activate mTORC1 signaling via promoting glutaminolysis [72]. Glutamine contributes to mTORC1 activation by being exchanged for leucine to stimulate mTORC1, which stimulates glutaminolysis in return by repressing SIRT4 and thus promotes the conversion from glutamate to α-KG [29, 73]. Consistent with our results, inhibition of GLS1 reduced mTORC1 signaling in Th17 cells and in the intestines of IL-10−/− mice [30, 74]. Besides, we found that inhibition of mTORC1 signaling caused a decrease in Collagen I expression, suggesting the expression of myofibroblast phenotype being sensitive to the regulation of mTOR signaling. mTORC1 inhibitor rapamycin was reported to inhibit the TGF-β-induced differentiation of corneal myofibroblasts, and ameliorated renal fibrosis in lupus nephritis by decreased anti-dsDNA antibody triggered downstream inflammatory and fibrotic processes [33, 75]. mTORC1 is also an essential component in the energy pathways, regulating mitochondrial functions. Previous studies have shown that mTOR activation increased the expression of oxidative metabolism genes and mitochondrial DNA copy number, whereas mTOR inhibition decreased mitochondrial oxidative function through a YY1-PGC-1alpha transcriptional complex [76, 77]. Our results suggested that glutaminolysis-related mTORC1 pathway participated in the acquisition of myofibroblast phenotype and maintenance of mitochondrial respiratory function during ESCs activation, and we further speculated that mTORC1 pathway was involved in the development of endometrial fibrosis in vivo [4, 78]. Subsequent animal studies and clinical samples would be needed to verify whether glutaminolysis participates in endometrial fibrosis and IUA in vivo through mTORC1 pathway.

## Conclusion

Our studies suggested that GLS1-mediated glutaminolysis was associated with ESCs activation, which played a crucial role in the development of endometrial fibrosis (Fig. 8). Glutaminolysis participated in energy supply and maintenance of mitochondrial function during ESCs activation. Glutaminolysis inhibition by BPTES intervention alleviated endometrial fibrosis and improved pregnancy outcomes in mice models. Mitochondria-related glutaminolysis pathway, mediated via mTOR signaling, suggested a novel mechanism of endometrial fibrosis and might be a potential therapeutic target for IUA.

## Materials and methods

### Patients and samples collection

This study was approved by the Human Ethics Committee of the Women’s Hospital, School of Medicine, Zhejiang University (20210227). Human tissues were obtained with written informed consent from each patient. A total of 31 menstruating women were employed in this study, excluding patients who had diabetes mellitus, hypertension, autoimmune disorder, other severe medical diseases, or received hormonal medication within six months before surgery. We obtained endometrial fibrous lesions from 5 women (mean age: 30.60 ± 4.39 years old, BMI: 20.57 ± 1.45) diagnosed with moderate and severe IUAs (Grade III–V, according to the American Fertility Society classification) by hysteroscopy. Normal endometrial tissues were obtained from 26 women (mean age: 30.28 ± 4.32 years old, BMI: 20.78 ± 2.32) undergoing hysteroscopic examination for hydrosalpinx or primary infertility. All specimens were in the proliferative phase of the cycle, which were identified by tissue histological examination. There were no statistical differences regarding age and BMI between the two groups. Endometrial tissues were fixed with 4% paraformaldehyde for histological analysis or transferred to the sterile DMEM/F12 medium for culture.

### Cell culture and cell treatment

Primary ESCs were obtained from human normal endometrial tissues as previously described [79]. Briefly, tissues were washed with sterile PBS and then cut into small pieces, followed by digestion with collagenase type I (0.2%, Sigma) on an orbital shaker for 1 h at 37 °C until the tissue was fully dissolved. The digestion suspension was filtered through a 100-µm cell strainer and then a 40-µm cell strainer. The obtained ESCs were resuspended in DMEM/F12 (Genom) containing 10% FBS (BioInd) and incubated in 5% CO_2_ at 37 °C. The medium was changed after 24 h. The purity of ESCs (> 95%) was confirmed by immunofluorescence using Vimentin, in keeping with the previous studies reported by our research group [79, 80]. The subsequent experiments were performed on ESCs at passages 3–8.

To construct a model of activation, ESCs were treated with 10 ng/ml TGF-β1 (PeproTech) for 48 h after overnight serum starvation. We added exogenous glutamine (Gibco) to glutamine-free DMEM medium (Gibco) to make glutamine-containing medium. For glutamine deprivation, cells were plated in glutamine-free DMEM medium, while non-glutamine-deprivation cells for comparison were cultured in glutamine-containing medium (glutamine-free DMEM medium supplemented exogenously with 2 mM glutamine).To explore the relationship between glutaminolysis and ESCs activation, ESCs were treated with TGF-β1, culturing in medium with or without 2 mM glutamine (Gibco), or pretreated with GLS1 inhibitors BPTES (10 µM, Selleck) or its vehicle (0.1% dimethyl sulfoxide, DMSO, Sigma) for 6 h. 2 mM α-Ketoglutarate (α-KG, Sigma) was used to supplement the metabolic intermediates in ESCs. In mTOR signaling experiment, ESCs were pretreated with a gradient of concentrations of Rapamycin (mTOR inhibitor, Selleck) for 12 h.

### RNA extraction, RNA sequencing and quantitative real-time PCR

Total RNA was isolated from ESCs lysates using the RNAiso Plus reagent (Takara Bio) and then reverse-transcribed using the PrimeScript Reverse Transcription reagent kit (Takara) according to the manufacturer’s instructions.

After the construction of an RNA-seq library, mRNA sequencing using the Illumina platform was carried out by OE biotech Co., Ltd. (Shanghai, China). Differential gene expression analysis was performed using the R package DEGseq software. The gene ontology (GO) enrichment analysis, and the Kyoto Encyclopedia of Genes Genomes (KEGG) pathway enrichment analysis were performed using the “phyper” function in the R software.

Quantitative real-time PCR was conducted using the SYBR Premix Ex TaqTM kit (Takara) with an Applied Biosystems 7900 HT system (Applied Biosystems). The average cycle threshold (Ct) was determined from triplicate wells, and the relative fold-change was determined using the 2^−ΔΔCt^ method. Specific primers used for amplification were synthesized by Tsingke (Beijing, China), the sequences of which were listed:

5′-AGGTCGGTGTGAACGGATTTG-3′ (forward) and.

5′-TGTAGACCATGTAGTTGAGGTCA-3′ (reverse) for GAPDH,

5′-AGGTGGTGATCAAAGGCATTC-3′ (forward) and.

5′- GCTTTTCTCTCCCAGACTTTCC-3′ (reverse) for GLS1,

5′-TCCTCATCCTCCCTTGAGAA-3′ (forward) and.

5′-ATGAAGGATGGCTGGAACAG-3′ (reverse) for α-SMA,

5′- GAGGGCCAAGACGAAGACATC-3′ (forward) and.

5′-CAGATCACGTCATCGCACAAC-3′ (reverse) for COL1A1.

### Protein extraction and western blotting

Protein extraction was prepared from ESCs using RIPA buffer (Beyotime Biotechnology) with proteinase and phosphatase inhibitors. After being quantified by the BCA assay kit (Thermo Scientific), equal amounts of proteins were separated by SDS-PAGE gel electrophoresis and transferred to polyvinylidene difluoride membranes (Merck Millipore). Membranes were blocked in 5% BSA (Sigma) at room temperature for 1 h, followed by incubation with primary antibodies against GAPDH (1:10000; Proteintech, 60004-1-Ig), GLS1 (1:1000; Abcam, ab93434), Collagen I (1:1000; Abcam, ab34710), α-SMA (1:1000; Abcam, ab7817), MFN1(1:1000; Abcam, ab57602), MFN2(1:1000; Abcam, ab56889), DRP1(1:1000; Abcam, ab56788), PINK1(1:1000; Cell Signaling Technology, 6946), p-p70-S6K (1:1000; Cell Signaling Technology, 9234), p-4E-BP1 (1:1000; Cell Signaling Technology, 2855) and p-S6 (1:1000; Cell Signaling Technology, 4858) overnight at 4 °C. The membrane was then incubated with HRP-conjugated goat anti-rabbit (1:10000; Proteintech, SA00001-2) or goat anti-mouse (1:10000, Proteintech, SA00001-1) secondary antibodies at room temperature for 1 h. Pageruler (ThermoFisher, 26,616) was used as the molecular marker. Protein expression was visualized with an ECL detection kit (Sigma-Aldrich). GAPDH was used as the loading control.

### Immunocytofluorescence

Immunofluorescence were performed in TGF-β1-treated ESCs to detect the co-localization of GLS1 and α-SMA. ESCs were seeded on 35 mm confocal dishes (Cellvis) at a density of 1 × 10^4^ cells/mL. After treatments, cells were fixed with 4% PFA for 15 min, permeabilized with 0.2% Triton X for 10 min and then blocked with 5% BSA for 45 min at room temperature. Primary antibodies α-SMA (1:200; Abcam, ab7818), GLS1 (1:200; Abcam, ab93434) were used for overnight incubation at 4 °C. DyLight488-conjugated goat anti-rabbit antibody (1:200; Multisciences, GAR4882) and DyLight594-conjugated goat anti-mouse antibody (1:200; Multisciences, GAM5942) were used for detection. DAPI was used to stain cell nucleus, and the stained ESCs were observed by an Olympus FV1200 confocal microscope (Olympus).

### Mice and treatment

Animal experiments were approved by the Institutional Animal Care and Use Committee (IACUC) of Zhejiang Chinese Medical University (IACUC-20200824-08). A total of 20 ICR female mice and 10 ICR male mice aged 8-weeks (BK Experimental Animal, Shanghai, China) were used for the experiments. All animals were housed and bred under standard recommended conditions in the animal research center of Zhejiang Chinese Medical University according to the institutional guidelines for laboratory animals.

Unilateral ethanol injection was performed to establish a murine IUA model, which was marked as day 0. After anesthesia with an intraperitoneal injection of 8% chloral hydrate (0.1  mL/10 g), each female mouse was subjected to an abdominal incision to expose the uterus. 95% ethanol was injected into one of its uterine horns, followed by repeated washing with saline 3 min later. The contralateral horn was injected with saline as non-injured controls. All unilateral injury female mice were then randomly divided into two groups (one DMSO-treated control group and one BPTES-treated group), with 10 females in each group. Treatment started from day 1 by intraperitoneal injection with 12.5 mg/kg BPTES (Selleck, S7753) or its vehicle (5% DMSO in PBS) once daily for 7 consecutive days. On day 8, five mice of each group were randomly selected and sacrificed to collect uteri for histological analysis, while the remaining females (5 females in each group) were mated with fertile males (1:1). The females were checked for the presence of a vaginal plug in the morning during the period of mating. Pregnancy was defined by the discovery of a vaginal plug. The mice were sacrificed after 14 days gestation to collect uterus and count the number of embryo implantation sites. The relative embryo implantation rates were calculated as the number of embryo implantation sites of the injured uterus/the number of embryo implantation sites of the uninjured uterus.

### Hematoxylin and eosin (HE) staining and Masson’s trichrome dye

HE staining and Masson’s trichrome staining were performed on endometrial samples from patients with IUA (*n* = 5) or without IUA (*n* = 5), and uterine samples from ICR mice. Average endometrial thickness of ICR uterine was measured under HE staining by Image-Pro Plus 6.0 software.

For fibrosis analysis, tissue sections were stained with Masson’s trichrome dye. Briefly, 4% paraformaldehyde-fixed, paraffin-embedded tissues were cut into 4-µm sections, followed by deparaffinization and rehydration, stained sequentially with Weigert’s iron haematoxylin solution, ponceau-acid fuchsin solution, phosphomolybdic acid solution and aniline blue solution. Collagenous fibers were dyed blue, while other components were stained red. Each section was randomly observed for five fields of the endometrium, and the blue area percentages of the endometrium were measured by Image-Pro Plus 6.0 software.

### Immunohistochemistry (IHC) and Immunohistofluorescence

To explore the relationship of fibrosis and glutaminolysis, endometrial samples from patients and uterine samples from ICR mice were used to detect the expression of αSMA and GLS1 by IHC and immunohistofluorescence. After deparaffinization and rehydration, paraffin-embedded Sect. (4-µm thick) were incubated with 3% hydrogen peroxide for 10 min to block endogenous peroxidase activity and heated in citrate (pH 6.0, > 95 ℃, 10 min) for antigen retrieval. Blocking was performed using 5% bovine serum albumin (BSA, Solarbio) for 45 min at room temperature. Sections were then incubated with primary antibodies: α-SMA (1:200; Abcam, ab7818); GLS1 (1:150; Abcam, ab93434) at 4 °C overnight.

For IHC staining, sections were then stained with secondary antibodies (DAKO) for 1 h, and diaminobenzidine (DAKO) was used for detection as a chromogen. Slices were analyzed under a microscope (Leica LMD). A semi-quantitative analysis of IHC was performed to measure the staining intensity according to the previous publication [79]. Briefly, each section was observed in five different fields, and we measured mean optical density (MOD) using Image-Pro Plus 6.0 software.

For immunohistofluorescence staining, sections were stained with DyLight488-conjugated goat anti-mouse antibody (1:200; Multisciences, GAM4882) or DyLight594-conjugated goat anti-rabbit secondary antibody (1:200; Multisciences, GAR5942) for detection. We used DAPI (Abcam) for cell nuclear staining. Representative images were obtained by an Olympus FV1200 confocal microscope (Olympus).

### MitoTracker staining

MitoTracker staining was used to observe the mitochondrial morphology in TGF-β1-treated ESCs. ESCs grown on 35 mm confocal dishes (Cellvis) were stained with 100 nM MitoTracker Green FM (Invitrogen) for 30 min at 37 °C. After washing three times with HBSS, mitochondrial morphology was observed using an Olympus FV1200 confocal microscope (Olympus). Hoechst 33342 (Beyotime) was used for nuclear staining. The Mitochondrial Network Analysis (MiNA) toolset, a combination of different ImageJ macros, was used for the semi-automated analysis of mitochondrial networks of cells [81]. By analyzing how pixels are spatially related, the toolset is defined to measure the length of each branch [82]. Three independent experiments were conducted, and 5 random fields of cells in each group were evaluated.

### Transmission electron microscopy (TEM)

Approximately 4 × 10^6^ ESCs were collected and fixed in 2.5% glutaraldehyde at 4 °C overnight and then fixed in 1% osmic acid at room temperature for 1 h. Samples were then fixed with 2% uranium acetate for 30 min, followed by gradient dehydration with 50%, 70%, 90%, 100% ethanol solution and 100% acetone solution for 15 min, respectively. After embedding, the samples were cut into 70 nm ultrathin sections and stained with 1% uranyl acetate and lead citrate for analysis under a transmission electron microscope (FEI Tecnai T10). The ultrastructure of mitochondria was observed at a magnification of ×4800 and ×49,000. Mitochondrial length analysis was performed as reported previously [82]. Experiment was repeated at least three times independently. Ten cells per group and 12–15 mitochondria per cell were measured randomly by Fiji software (Image J version 2.0.0-rc-69/1.52p).

### Mitochondrial membrane potential (MMP, ΔΨm)

As a critical indicator of mitochondrial function, mitochondrial membrane potential (MMP) is detected. JC-10 mitochondrial membrane potential staining kit (MAK160, Sigma) was used according to the manufacturer’s instructions. The JC-10 dye loading solution was prepared by adding 25 µL of 200 × JC-10 to 5 mL of assay buffer. Cells were then incubated with 500 µL of JC-10 loading solution protected from light at 37 °C for 30 min, followed by washing with wash solution. Untreated cells incubated with 5 µM FCCP for 30 min before JC-10 incubation were set as positive controls. JC-10 forms J-aggregates, which generates red fluorescence at high Δψm, while remains in the monomeric form at low Δψm. Fluorescence intensity of aggregates and of monomeric forms were detected by a flow cytometer (Cytoflex, Beckman, USA), and the relative ratio of red and green fluorescence reflect the level of mitochondrial membrane potential.

#### Measurement of mitochondrial ROS levels

Mitochondrial ROS was detected by the MitoSOX Red probe (MedChemExpress, HY-D1055) according to the manufacturer’s instructions. ESCs cultured in 6-well dishes were stained in situ with 1 µM MitoSOX Red staining solution for 30 min at 37 °C. After washing three times with serum-free medium, the stained cells from each group (*n* = 5) were observed using a fluorescent microscope (EVOS M500, Thermo Scientific) at an excitation/emission wavelength of 510/580 nm. 5 random fields of cells in each well were evaluated, and the mean fluorescence intensity was analyzed by Image-Pro Plus 6.0 software.

### Mitochondria isolation and ATP measurement

Mitochondria were isolated using Cell Mitochondria Isolation Kit (Beyotime, C3601) following the manufacturer’s instructions. Briefly, cells from each group (*n* = 5) were collected and resuspended in the mitochondria isolation buffer, followed by incubation on ice for 10 min. Then the solution was homogenized for about 10 times until trypan blue staining indicated that over 50% of the cells were positive, followed by centrifugation at 600*g* for 10 min at 4 °C. The resulting supernatants were collected and centrifuged at 11,000*g* for 10 min at 4 °C, and then isolated mitochondria were obtained by discarding the supernatants.

The isolated mitochondria were resuspended in respiration buffer (composed of 250 mM sucrose, 15 mM KCl, 1 mM EGTA, 30 mM K_2_HPO_4_, 5 mM MgCl_2_, pH 7.4, as previously reported [83]), followed by incubation at 37 °C for 20 min after supplementation with succinate (25 mM final concentration) and adenosine diphosphate (ADP, 1.65 mM final concentration). The respiration buffer containing mitochondria was then centrifuged at 10,000 g for 10 min, and supernatants were used for the ATP measurements [84]. Briefly, 100 µL of the supernatants and 100 µL of Celltiter-Glo regeant (from the Celltiter-Glo luminescent cell viability assay kit, Promega G7570) were added per well in a 96-well white plate. After stabilization at room temperature for 10 min, the luminescent signal was determined immediately using the Varioskan® Flash Multimode Reader (Thermo Scientific). Mitochondrial ATP concentration was calibrated with the ATP standard solution (Beyotime, D7378). The mitochondrial protein concentration was determined using a bicinchoninic acid (BCA) assay kit (Beyotime, P0012S), and the mitochondrial ATP levels were standardized by protein concentration.

#### Measurement of the oxygen consumption rate (OCR)

To assess mitochondrial respiratory functions, OCR was measured using the Seahorse XF96 Analyzer (Seahorse Bioscience) and the Seahorse XF Cell Mito Stress Test Kit (Seahorse Bioscience) according to the manufacturer’s instructions. ESCs were seeded in Seahorse 96-well microplates at a density of 10,000 per well and cultured at 37 °C with 5% CO_2_. 1 h prior to the OCR measurement, cell culture media was replaced with Seahorse assay medium, in which cells were incubated at 37 °C without 5% CO_2_. OCR was measured at baseline and following sequential injections of 1.5 mM oligomycin (ATP synthase inhibitor), 2 mM FCCP (OXPHOS uncoupler), and 0.5 mM rotenone/antimycin A (respiratory chain complex I and III inhibitor). Maximal respiration was calculated as FCCP-induced respiration. ATP-linked OCR was defined as oligomycin–inhibited OCR. Spare capacity was calculated subtracting basal respiration from maximal respiration [79]. To measure acute responses to glutamine, cells were cultured in glutamine-free medium at first, and glutamine was re-added during the measurement of OCR.

### Statistical analysis

All experiments were repeated independently at least three times. The data were analyzed using GraphPad Prism 8.0 software and were expressed as the mean ± standard error of mean (SEM). Normality of the data was assessed based on Shapiro–Wilk normality test prior to statistical calculation. Statistical analysis between two groups was performed using the appropriate parametric Student’s t test or nonparametric Mann–Whitney test. Comparisons of multiple groups for parametric data were performed by one-way analysis of variance (ANOVA) followed by Turkey’s multiple comparisons test (for pairwise comparisons between groups). A Kruskal–Wallis test and Dunn post hoc were used for nonparametric data. For the results compared with their control (set at 1), statistical analysis was analyzed using a one-sample Student’s t-test, and subsequent comparisons of treatment groups was performed using the appropriate parametric or nonparametric analysis depending on the normality of data [85–87]. *p* < 0.05 was considered statistically significant.

## Data Availability

The datasets used or analysed during the current study are available from the corresponding author on reasonable request.

## Abbreviations

α-KG: α-ketoglutarate
α-SMA: Alpha-smooth muscle actin
ADP: Adenosine diphosphate
ANOVA: Analysis of variance
ATP: Adenosine Triphosphate
BPTES: Bis-2-(5-phenylacetamido-1,2,4-thiadiazol-2-yl) ethyl sulfide
BSA: Bovine serum albumin
DMSO: Dimethyl sulfoxide
DRP1: Dynamin-related protein 1
ECM: Excessive deposition of extracellular matrix
EGTA: Ethylene Glycol Tetraacetic Acid
ESCs: Endometrial stromal cells
Gln: Glutamine
Glu: Glutamate
GO: Gene ontology
GOT2: Glutamate-oxaloacetate transaminase 2
GPT2: Glutamate-pyruvate transaminase 2
GLS1: Glutaminase1
GLUD1: Glutamate dehydrogenase1
HE: Hematoxylin and eosin
HIF-1α: Hypoxia-inducible factor-1α
IHC: Immunohistochemistry
IUA: Intrauterine adhesion
KEGG: Kyoto Encyclopedia of Genes Genomes
MFN1: Mitofusin 1
MFN2: Mitofusin 2
MiNA: Mitochondrial network analysis
MMP: Mitochondrial membrane potential
MOD: Mean optical density
mTOR: Mammalian target of rapamycin
mTORC1: mTOR complex 1
OCR: Oxygen consumption rate
OXPHOS: Oxidative phosphorylation
p70-S6K: p70 ribosome S6 kinase
PINK1: PTEN-induced putative kinase 1
Rapa: Rapamycin
ROS: Reactive oxygen species
RT: Room temperature
TCA: Tricarboxylic acid
TEM: Transmission electron microscopy
TGF-β1: Transforming growth factor-β1
